# The Effect of Visual Apparent Motion on Audiovisual Simultaneity

**DOI:** 10.1371/journal.pone.0110224

**Published:** 2014-10-08

**Authors:** Jinhwan Kwon, Ken-ichiro Ogawa, Yoshihiro Miyake

**Affiliations:** Department of Computational Intelligence and Systems Science, Tokyo Institute of Technology, Yokohama, Kanagawa, Japan; University of Montreal, Canada

## Abstract

Visual motion information from dynamic environments is important in multisensory temporal perception. However, it is unclear how visual motion information influences the integration of multisensory temporal perceptions. We investigated whether visual apparent motion affects audiovisual temporal perception. Visual apparent motion is a phenomenon in which two flashes presented in sequence in different positions are perceived as continuous motion. Across three experiments, participants performed temporal order judgment (TOJ) tasks. Experiment 1 was a TOJ task conducted in order to assess audiovisual simultaneity during perception of apparent motion. The results showed that the point of subjective simultaneity (PSS) was shifted toward a sound-lead stimulus, and the just noticeable difference (JND) was reduced compared with a normal TOJ task with a single flash. This indicates that visual apparent motion affects audiovisual simultaneity and improves temporal discrimination in audiovisual processing. Experiment 2 was a TOJ task conducted in order to remove the influence of the amount of flash stimulation from Experiment 1. The PSS and JND during perception of apparent motion were almost identical to those in Experiment 1, but differed from those for successive perception when long temporal intervals were included between two flashes without motion. This showed that the result obtained under the apparent motion condition was unaffected by the amount of flash stimulation. Because apparent motion was produced by a constant interval between two flashes, the results may be accounted for by specific prediction. In Experiment 3, we eliminated the influence of prediction by randomizing the intervals between the two flashes. However, the PSS and JND did not differ from those in Experiment 1. It became clear that the results obtained for the perception of visual apparent motion were not attributable to prediction. Our findings suggest that visual apparent motion changes temporal simultaneity perception and improves temporal discrimination in audiovisual processing.

## Introduction

In this study, we address the integration of multisensory temporal information from the environment. Visual motion information from a dynamic environment is an especially influential factor in temporal perception. However, it is unclear how visual motion information influences temporal perception. We investigated whether visual apparent motion, which provides a good representation of the specific characteristics of motion perception, affects audiovisual temporal perception.

Temporal perception is an important topic in the study of multisensory integration [1]. There has been remarkable progress in simultaneity judgment (SJ) and temporal order judgment (TOJ) tasks in psychophysical studies that investigate temporal factors in multisensory integration [2], [3], [4], [5]. In particular, TOJ tasks are a known way to measure human perception of temporal asynchrony between two or more senses. In this method, the point of subjective simultaneity (PSS) and just noticeable difference (JND) are two important parameters. The PSS represents the interval between the applications of stimuli to two senses at which both are perceived by the senses as occurring at the same time. The JND can be used as an indicator of temporal resolution in cross-modality [6].

Many studies have shown that audition dominates vision in the time dimension [7], [8], [9], [10], [11], [12], [13]. In particular, auditory driving, whereby audition captures vision, is a widely known phenomenon [7], [8]. For example, Fendrich and Corballis (2001) reported that a flash was perceived significantly earlier when preceded by an auditory stimulus [8]. On the other hand, a flash was seen significantly later when followed by an auditory stimulus [8]. In TOJ tasks using a set of audio and visual stimuli, many studies have demonstrated that hearing changes or attracts visual temporal perception [9], [10]. For example, Morein-Zamir et al. (2003) investigated whether auditory events can alter the timing of visual events through a visual TOJ task [10]. As a result, TOJ performance was improved with a smaller JND for auditory stimuli when one auditory stimulus was presented shortly before the first light and another after the second light [10]. Although many studies find that PSSs and JNDs depend on a variety of multisensory information in TOJ tasks [14], [15], [16], [17], [18], [19], it remains unclear how motion information influences audiovisual temporal perception. Motion information is an important factor in temporal integration during multisensory processing. Therefore, there is a need for quantitative investigation of the influence of motion information on the integration of multisensory temporal perception.

In this study, we focused on visual apparent motion. Visual apparent motion is an optical phenomenon whereby motion appears to occur at a certain spatiotemporal interval, although two discrete stimuli are used [20], [21], [22], [23], [24]. In particular, visual apparent motion is systematically affected by the temporal interval between two stimuli. When the temporal interval is too short, the stimuli are perceived as simultaneous, whereas when the temporal interval exceeds a certain interval, the two stimuli are perceived as successive. Therefore, if the temporal interval between two stimuli is too short or too long, motion is not perceived. For example, many researchers have reported that two visual stimuli are perceived as a continuous motion when the interstimulus onset interval (ISOI) of the visual stimuli is within a range of 50 to 150 ms. Conversely, the visual stimuli are perceived as successive beyond an ISOI of 300 ms [20], [25], [26], [27], [28]. Of particular note is that apparent motion is a fundamental unit of visual motion in finite time.

The purpose of the present study is to investigate how visual apparent motion affects audiovisual temporal perception. We examined three types of TOJ task experiments. In Experiment 1, we examined whether visual apparent motion has an effect on an audiovisual TOJ task. Participants conducted audiovisual TOJ tasks in the apparent motion condition with two flashes, and in the normal condition with a single flash, which is the conventional condition of a TOJ task. However, it was insufficient only to compare the apparent motion condition with the normal condition because the two conditions in Experiment 1 may differ in the amount of visual stimulation available. Therefore, there was a need to examine the effect of visual apparent motion with identical amounts of visual stimulation. In Experiment 2, we examined two kinds of TOJ tasks in the apparent motion condition and in the successive condition. In previous studies, when the temporal interval between two flashes was long, visual apparent motion was not detected; the two flashes were perceived as successive events with no movement [25], [26], [27]. Moreover, there may remain an influence not only of apparent motion, but also of specific prediction as a higher-order brain function, because the interval between the two flashes in the apparent motion condition was constant. Therefore, it was necessary to conduct a supplementary experiment in which the interval was changed randomly. In Experiment 3, we eliminated the influence of prediction by randomizing the intervals between the two visual stimuli.

## Materials and Methods

### Task Designs

We conducted audiovisual TOJ tasks in three experiments in which the spatial location and duration of stimuli were identical. In Experiment 1, we examined whether visual apparent motion had an effect on an audiovisual TOJ task. Participants performed the TOJ task in the apparent motion condition in which two flashes were presented with a stimulus onset asynchrony (SOA) of 137 ms [26], and in the normal condition with a single flash. In Experiment 2, we investigated the influence of the amount of visual stimulation. For that reason, we set up two kinds of TOJ tasks in the apparent motion condition with an SOA of 137 ms, which is the same spatiotemporal interval as in Experiment 1, and in the successive condition with SOAs of 300 and 500 ms between the two flashes, which are perceived as successive stimuli without motion. In Experiment 3, we wished to eliminate the effect of prediction because of constant intervals, and therefore, we presented the two visual stimuli with SOAs of 137, 300, or 500 ms in random order.

### Participants

Eighteen participants (16 males and two females, with a mean age of 24.3 years) participated in Experiment 1. Twelve participants (10 males and two females, with a mean age of 23.9 years) took part in Experiment 2. Twelve participants (11 males and one female, with a mean age of 23.5 years) took part in Experiment 3. All participants had normal hearing and normal or corrected-to-normal visual acuity, and were naive as to the purpose of the experiment. Participants were paid to take part in the experiments, and written informed consent was obtained. These experiments were approved by the ethics committee of the Tokyo Institute of Technology.

### Apparatus and Stimuli

**Figure 1:**
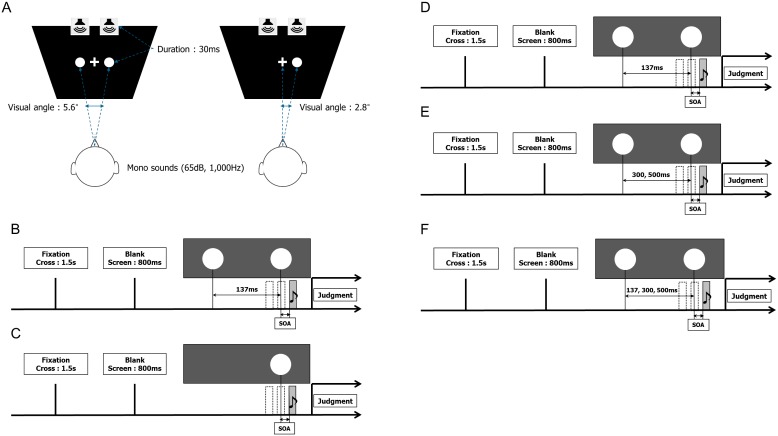
Schematic illustration of Experiments 1, 2 and 3. (A) shows the setup for the experiments. Participants sit in front of a monitor with loudspeakers placed on top and a fixation point of a white cross displayed in the center. The visual angle was 2.8° for the single stimulus and 5.6° for two stimuli. Sound stimuli were presented via the two speakers. (B) and (C) show the procedure for Experiment 1. After the presentation of a fixation cross (1.5 s) and a blank screen (800 ms), two visual stimuli were presented with an SOA of 137 ms (apparent motion condition: (B)), or a single visual stimulus was presented (normal condition: (C)). (D) and (E) show the procedure for Experiment 2. After the presentation of a fixation cross (1.5 s) and a blank screen (800 ms), two visual stimuli were presented in the apparent motion condition (D) and two visual stimuli with SOAs of 300 and 500 ms in the successive condition (E). (F) shows the procedure for Experiment 3. After the presentation of a fixation cross (1.5 s) and a blank screen (800 ms), two visual stimuli were presented with SOAs of 137, 300 and 500 ms in random order (random order condition). In all the experiments, sounds were presented either before or after the visual stimuli at SOAs ranging from –120 to 120 ms at 30 ms intervals in random order.

All TOJ task experiments were conducted in a dark and soundproof room (0.00–0.01 cd/m^2^ luminance). Figure 1A illustrates the setup for the experiments. Visual stimulation was provided by a 27-inch LCD display (Samsung S27A950D, Korea) with a screen resolution of 1920×1080 pixels and a refresh rate of 120 Hz. The display was operated from a PC workstation (Apple Mac Pro, 3.2 GHz Quad-Core Intel Xeon, ATI Radeon HD 5770 graphic card, 1 GB GDDR5 memory, Cupertino, CA, USA) placed in front of the participants. Their head position was fixed by a chin rest at a viewing distance of 100 cm. A white cross of 2 cm in length was displayed as a fixation point in the center of the screen. Visual stimuli consisted of one or two white disks of 3.2 cm in diameter on a black background. The luminance of the black background on the screen was 0.09 cd/m^2^, and that of the white disks was 74.8 cd/m^2^. The visual angle was 2.8° for the single stimulus and 5.6° for two visual stimuli. Sound stimuli were presented as monaural sounds (65 dB, 1,000 Hz) delivered via two speakers (MM-SPWD3BK, Sanwa Supply, Japan). The speakers were located on top of the screen. These visual and auditory stimuli were generated and operated with a computer program (Matlab and Psychtoolbox-3, MA, USA).

### Procedure

In Experiment 1, the participants sat on a chair facing the stimulus, and a constant head position was maintained by means of the chin rest. The audiovisual TOJ tasks were performed over two sessions with visual stimuliin the apparent motion condition and in the normal condition. Figures 1B and 1C illustrate the procedure for Experiment 1. In the apparent motion condition (Fig. 1B), each trial began with display of the fixation cross for 1.5 s, followed by a dark blank screen for 800 ms. Next, one white circle for the first visual stimulus was displayed for 30 ms, and the second stimulus was presented with an SOA of 137 ms for 30 ms. To assess the temporal discrimination of a pair of auditory and visual stimuli, one brief sound (30 ms) was presented at various times relative to the second visual stimulus. The participants were instructed to complete a TOJ task between the second visual stimulus and the brief sound. The onset time of the auditory stimulus paired with a visual stimulus was randomly selected from the following SOA values: –120, –90, –60, –30, 0, +30, +60, +90, and +120 ms (where the negative values indicate that the auditory stimulus preceded the visual stimulus). Then, after 500 ms, the participants made a forced-choice judgment with respect to the order of the audiovisual stimuli by answering the question “which one was first?” The answers consisted of “light first,” which was chosen by pressing the Z key, and “sound first,” which corresponded to the X key. The “light first” response was selected when the flash was ahead of the sound, and the “sound first” response was selected when the sound preceded the flash. In the normal condition (Fig. 1C), the first visual stimulus was not presented. That is, only the second stimulus in the apparent motion condition was shown in this session, and the other process was the same as that in the TOJ task in the apparent motion condition. The same method of evaluating the temporal discrimination between sound and flash with the same SOA values was then used as in the apparent motion condition. Experiment 1 consisted of 270 trials (2 visual conditions ×9 audiovisual SOAs×15 repetitions) in counterbalanced order and the experiment was divided into 10 blocks of 27 trials (9 audiovisual SOAs×3 repetitions). Including practice for each task, each experiment took approximately one and a half hours.

In Experiment 2, the procedure was the same as in Experiment 1 with the following exceptions. Experiment 2 consisted of two kinds of TOJ tasks in the apparent motion condition (Fig. 1D) and the successive condition (Fig. 1E). The apparent motion condition was equivalent to that of Experiment 1, with an SOA of 137 ms between the two flashes. The successive condition consisted of SOAs of 300 or 500 ms between the two flashes. The timing of the auditory stimulus relative to the second flash was the same as in Experiment 1. The participants were instructed to judge the order of the second visual stimulus and the brief sound. Experiment 2 consisted of 405 trials (3 visual conditions ×9 audiovisual SOAs×15 repetitions) in counterbalanced order and the experiment was divided into nine blocks of 45 trials (9 audiovisual SOAs×5 repetitions). Including practice for each task, each experiment took approximately one and a half hours.

In Experiment 3, the procedure was the same as in Experiment 1, with the following exceptions. In Experiment 3, to confirm the influence of prediction with constant intervals, the participants conducted the TOJ tasks with SOAs of 137 ms, 300 ms, and 500 ms presented in random order between the visual stimuli (Fig. 1F). The timing of the auditory stimulus relative to the second flash was the same as in Experiment 1. The participants were instructed to judge the order of the second visual stimulus and the brief sound. Experiment 3 consisted of 432 trials (3 visual conditions ×9 audiovisual SOAs×16 repetitions) in counterbalanced order and the experiment was divided into eight blocks of 54 trials (3 visual conditions ×9 audiovisual SOAs×2 repetitions). Including practice for each task, each experiment took approximately one and a half hours.

Before starting each experiment, we examined whether the participants perceived motion between two flashes. In Experiment 1, we showed the participants two flashes with an SOA of 137 ms 10 times, and participants were asked to evaluate whether their impression of the stimuli was of “continuous motion” or “successive stimuli.” In Experiments 2 and 3, we showed them two flashes, with SOAs of 137 ms, 300 ms and 500 ms, 10 times in counterbalanced order. Then, the participants were asked to evaluate whether their impression of the stimuli was of “continuous motion” or “successive stimuli.” Only the participants who perceived “continuous motion” with an SOA of 137 ms and “successive stimuli” with SOAs of 300 ms and 500 ms for all stimuli continued to participate in the experiment. We confirmed that motion was perceived during the TOJ task after each experimental session was completed.

### Data Analysis

The ratio of answers indicating earlier presentation of an auditory stimulus was calculated for each SOA. We conducted logistic regressions using a generalized linear model with the ratio data from each experiment [29]. The following was applied in the regression analysis:
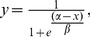
(1)where *α* represents estimated PSS, *x* denotes SOA, and 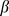 is related to JND. JND is calculated as shown in the following equation
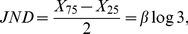
(2)where 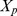 represents the SOA with 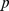 percent of “auditory first” responses.

**Figure 2:**
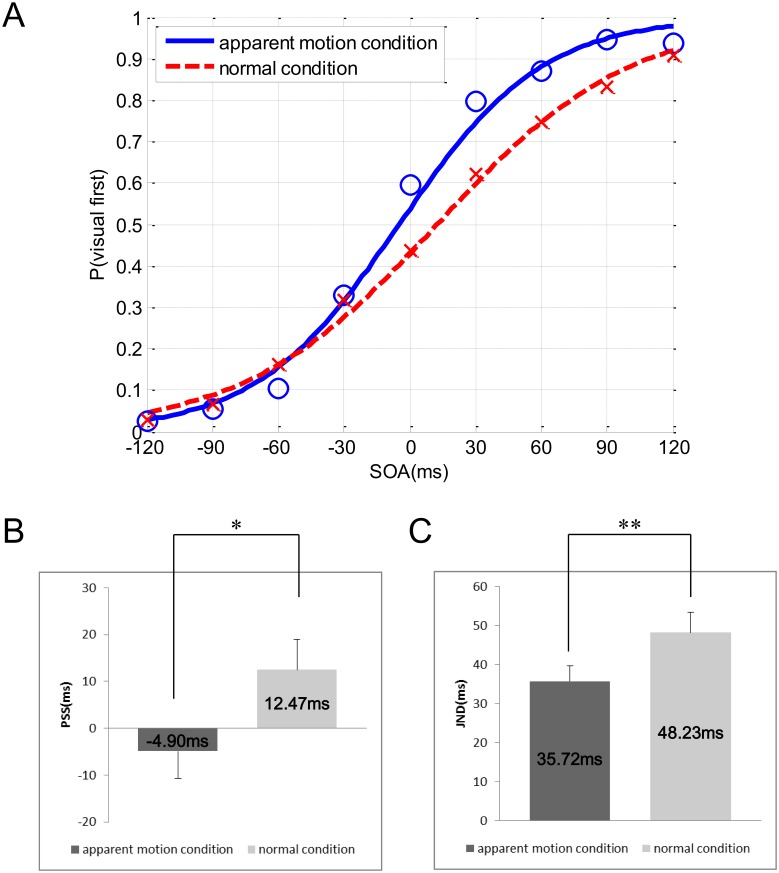
Results of Experiment 1. (A) shows psychometric curves fitted to the distribution of mean TOJ data for all participants in the apparent motion condition and in the normal condition. The negative values of the SOAs indicate that an auditory stimulus preceded a visual stimulus. (B) shows the mean PSSs in the apparent motion and normal conditions. Error bars represent the standard error of the means. (C) shows the mean JNDs in the apparent motion and normal conditions. Error bars represent the standard error of the means, *: *p*<.05, **: *p*<.01, for a paired t-test.

As Figure 2A illustrates, psychometric curves were fitted to the distribution of the mean TOJ data for each condition. We determined the JND and PSS values for each participant using regression analysis (Equations (1) and (2)) and calculated mean and standard error values from the data.

## Results

### Experiment 1

The results of two participants were excluded because they did not perceive continuous motion. Figure 2 shows the results of Experiment 1. Figure 2A illustrates psychometric curves fitted to the distribution of the mean TOJ data for all participants in the apparent motion and normal conditions. As shown in Figure 2B, the PSS in the normal condition had a positive value of 12.47 ms (SE = 6.45), but the PSS in the apparent motion condition shifted toward a sound-lead stimulus of –4.90 ms (SE = 5.84). This result indicates that a pair of audiovisual stimuli was perceived simultaneously when the auditory stimulus preceded the visual stimulus. A paired t-test of PSSs indicated a significant difference between the TOJ tasks in the apparent motion condition and those in the normal condition (t(15) = –2.33, *p* = 0.034, see Table S1). In addition, the JND in the apparent motion condition was smaller than that in the normal condition (see Fig. 2C), and the JND values were 35.72 ms (SE = 3.96) and 48.23 ms (SE = 5.17), respectively. A significant difference between the JNDs was observed in the paired t-test (t(15) = –3.57, *p* = 0.001, see Table S1).

### Experiment 2

**Figure 3:**
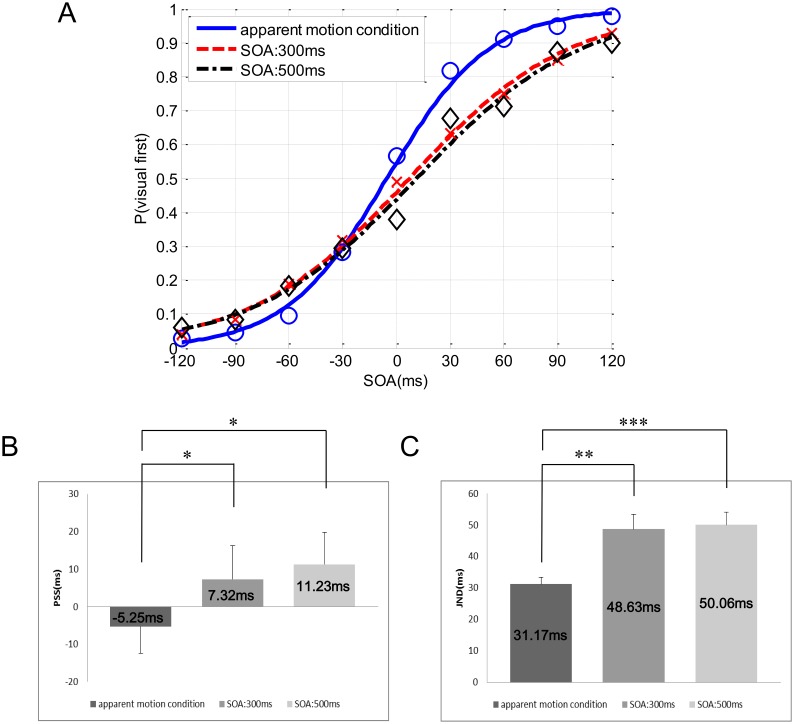
Results of Experiment 2. (A) shows psychometric curves fitted to the distribution of mean TOJ data for all participants in the apparent motion and successive conditions. The negative values of the SOAs indicate that an auditory stimulus preceded a visual stimulus. (B) shows the mean PSSs in the apparent motion and successive conditions. Error bars represent the standard error of the means. (C) shows the mean JNDs in the apparent motion and successive conditions (SOA: 300 ms and 500 ms). Error bars represent the standard error of the means. The *p* values were adjusted for multiple comparisons using Holm’s correction, *: *p*<.05, **: *p*<.01, ***: *p*<.001.

In Experiment 2, the participants performed both kinds of TOJ tasks in the apparent motion and successive conditions. Individual PSSs and JNDs for the two conditions were computed as in Experiment 1. Figure 3A illustrates the results of Experiment 2 with psychometric curves fitted to the distribution of the mean TOJ data of all participants in the apparent motion and successive conditions. Figures 3B and 3C show the results of PSS and JND in Experiment 2. The PSS shifted toward a sound-lead stimulus in the apparent motion condition, and the JND in the apparent motion condition was smaller than that in the successive condition. In particular, the PSS and JND in the apparent motion condition were almost identical to those obtained in the apparent motion condition in Experiment 1, and similar results were obtained in the successive condition in Experiment 2 and in the normal condition in Experiment 1. A repeated-measures analysis of variance (ANOVA) of the PSSs showed a significant main effect for the temporal interval, *F*(2, 23) = 15.83, *p*<0.001 (see Table S2). Multiple comparisons with Holm correction showed significant differences between the apparent motion condition and the successive condition (apparent motion and an SOA of 300 ms: *p* = 0.042, apparent motion and an SOA of 500 ms: *p* = 0.012, see Table S2). Moreover, a repeated-measures ANOVA of the JNDs revealed a significant main effect of the temporal interval, *F*(2, 23) = 25.03, *p*<0.001 (see Table S2). In addition, multiple comparisons with Holm correction confirmed that there was a significant difference between the apparent motion and successive conditions (apparent motion and an SOA of 300 ms: *p* = 0.002, apparent motion and an SOA of 500 ms: *p*<0.001, see Table S2).

### Experiment 3

**Figure 4:**
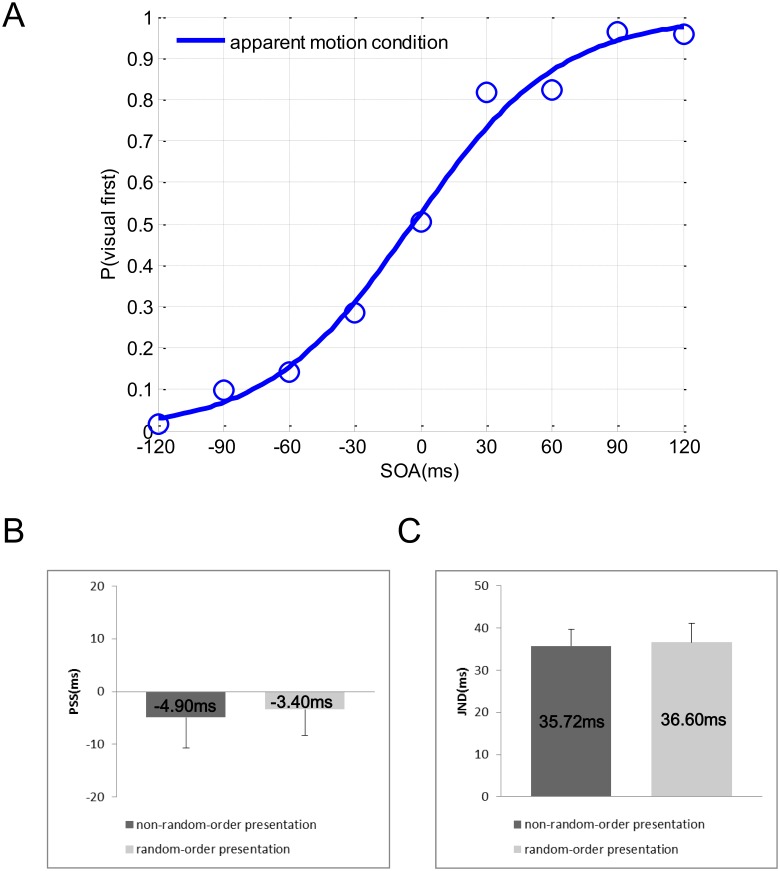
Results of Experiment 3. (A) shows psychometric curves fitted to the distribution of the mean TOJ value for all participants in the random order condition. The negative values of the SOAs indicate that an auditory stimulus preceded a visual stimulus. (B) shows the mean PSSs from presentation at nonrandom intervals (the apparent motion condition in Experiment 1) and random intervals (the apparent motion condition in Experiment 3). Error bars represent the standard error of the means. (C) shows the mean JNDs of the apparent motion and the random order conditions. Error bars represent the standard error of the means, *: *p*<.05, **: *p*<.01, unpaired t-test.

In Experiment 3, the participants performed the TOJ task with an interval between two visual stimuli that was varied in random order, and only the results obtained in the apparent motion condition (with an SOA of 137 ms) were extracted. All the participants perceived continuous motion, and the PSSs and JNDs were computed as in Experiment 1. Figure 4A shows the results of Experiment 3, and Figures 4B and 4C show the results for the PSSs and JNDs in Experiment 3. The random- and nonrandom-order presentations reflect the results of the apparent motion condition in Experiment 1. The values of the PSS and JND in the apparent motion condition in Experiment 3 were almost the same as those obtained in the apparent motion condition in Experiment 1. An unpaired t-test of PSSs and JNDs for the TOJ tasks in the apparent motion condition indicated no significant difference between Experiments 1 and 3 (t(26) = –0.11, *p* = 0.92, t(26) = –0.12, *p* = 0.91, see Table S3).

## Discussion

The results of Experiment 1 show that the PSS in the apparent motion condition was shifted toward a sound-lead stimulus, which differs from the PSS in the normal condition. Moreover, the JND in the apparent motion condition was smaller than that in the normal condition. In Experiment 2, the results obtained in the apparent motion condition differ from those obtained in the successive condition, which included the same amount of visual stimulation as the apparent motion condition. Similar results were obtained in the apparent motion conditions in Experiments 1 and 2, and the results obtained in the successive condition were similar to those obtained in the normal condition in Experiment 1. In particular, the results of Experiment 3, which eliminated the effect of prediction, were no different from those obtained in the apparent motion condition in Experiment 1. We use these results to clarify the influence of visual apparent motion on audiovisual simultaneity perception.

Visual apparent motion changes temporal simultaneity perception and improves temporal discrimination in audiovisual processing. With respect to temporal simultaneity, Experiment 1 shows that the PSSs in the normal condition are similar to those of previous studies, which were usually shifted toward a visual-lead stimulus [5], [6], whereas the PSSs in the apparent motion condition were shifted toward a sound-lead stimulus. Previous studies have been conducted using a simple set of stimuli, such as a combination of a single sound and a single flash in audiovisual simultaneity. For example, the temporal ventriloquism effect did not affect the baseline PSS. In the temporal ventriloquism effect condition, one auditory stimulus was presented shortly before the first light, and the other after the second light. In the baseline condition, each auditory stimulus was presented at the same time as a flash, (i.e., each pair of an auditory stimulus and a visual stimulus was presented at the same time) [10] (see Table 1). It has also been reported that the PSS of audiovisual simultaneity perception usually shifts toward a visual-lead stimulus, so maximal simultaneity is perceived if light comes slightly before sound [30], [31], [32], [33], [34], [35], [36]. Furthermore, several studies reported that the PSS changed under audiovisual simultaneity, in which the PSS became closer to physical simultaneity (i.e., zero). For instance, in audiovisual speech, the PSS of congruent audiovisual speech stimuli (the mean PSS of 23 ms) shifted closer to physical simultaneity than that of incongruent audiovisual speech under the McGurk effect (a mean PSS of 37 ms; see Table 1) [36]. Moreover, Zampini et al. (2003) reported that the PSS was closer to physical simultaneity when audiovisual stimuli were presented in the same spatial position than when they were presented in different spatial positions (see Table 1) [35]. This means that the same spatial positions of simple audiovisual stimuli and congruent utterance information in audiovisual speech shifted the PSS toward physical simultaneity in audiovisual integration, but the tendency toward visual-lead stimulus in the PSS did not change. However, in this study, visual apparent motion changed the PSS relative to the sound-lead stimulus, which is closer to physical simultaneity than in the abovementioned studies. This may mean that visual apparent motion contributes to very precise perceptions of temporal simultaneity, which is closer to physical simultaneity, in audiovisual integration.

**Table 1:** Changes in PSS and JND in audiovisual integration in previous studies.

	PSS		JND	
Temporal ventriloquism	baseline	AVVA	baseline	AVVA
	Not changed		62 ms	45 ms
Audiovisual speech	congruent	incongruent	congruent	incongruent
	23 ms	37 ms	205 ms	161 ms
Audiovisual spatial position	same	different	same	different
	60 ms	75 ms	32 ms	22 ms

Temporal ventriloquism: “baseline” means that each auditory stimulus was presented at the same time as a flash (i.e., each pair of audiovisual stimuli were presented simultaneously), and “AVVA” indicates that one sound was presented before the first flash and the other sound after the second flash. Audiovisual speech: “congruent” means that auditory speech stimuli were congruent with visual speech stimuli, and “incongruent” indicates that auditory speech stimuli were not congruent with visual speech stimuli, as in McGurk effect speech. Audiovisual spatial position: “same” means the same spatial location, and “different” indicates a different spatial location for audiovisual stimuli.

With respect to temporal resolution, we found that visual apparent motion resulted in greater temporal discrimination. The JND is regarded as an indicator of the temporal window of sensory integration, because it represents the resolution of temporal discrimination between the senses. The JND is known to be in the range of 30–60 ms in audiovisual TOJ tasks using a set of simple stimuli, such as a pair consisting of a single sound and single flash [10], [35], [37]. On the other hand, it has been reported that the JND in audiovisual speech is greater (a temporal window of approximately 200 ms) than that in the above-described TOJ tasks in audiovisual integration [36]. In particular, previous studies have reported that this temporal resolution changes according to a variety of factors such as spatial or temporal separation of stimuli and predictions regarding the presentation of stimuli [14], [15], [16], [17], [18], [19]. For example, the temporal ventriloquism effect shifted the JND toward smaller values than those of the baseline (see Table 1) [10]. On the other hand, in audiovisual speech, the JND of congruent audiovisual speech stimuli (a mean JND of 205 ms) is larger than that of incongruent audiovisual speech such as that under McGurk effect (with a mean JND of 161 ms; see Table 1) [36]. With respect to spatial separation, Zampini et al. (2003) reported that temporal resolution in audiovisual integration was improved when audiovisual stimuli were presented in different locations rather than in the same location (see Table 1) [35]. However, there remained a need to examine whether motion information influences temporal resolution in audiovisual processing. In this study, apparent motion shows greater temporal resolution than that which occurs in the normal condition. Therefore, visual apparent motion is a new factor that increases temporal discrimination.

Experiment 2 shows that the PSSs and JNDs in audiovisual temporal perception differed according to whether visual apparent motion was present or absent. In Experiment 1, it remained unclear whether the results were influenced by differences in the amount of visual stimulation between the apparent motion condition with two flashes and the normal condition with a single flash. Because visual energy increases in the apparent motion condition, there was a possibility that visual apparent motion influenced perceptions of audiovisual simultaneity. In addition, because double visual stimuli in the apparent motion condition would prime the visual processing system, this may change the relative perception of audiovisual simultaneity compared with the use of a single visual stimulus [38], [39]. It is particularly notable that Spence et al. (2001) reported that the temporal processing of modalities was affected not only by the prediction of modality expectancies but also by the quantity of modality-induced stimuli [38]. In Experiment 2, therefore, the amount of visual stimulation in the two conditions was equalized, and the participants then conducted TOJ tasks in the apparent motion condition and in the successive condition. However, the amount of visual stimulation made no difference in the apparent motion condition.

Experiment 3 shows that apparent motion was equivalently processed regardless of prediction. With respect to visual prediction and attention, it should be noted that when participants know the specific time at which targets appear, specific attention can be allocated [40]. Furthermore, it is known that predictable and anticipated information improves temporal resolution and temporal sensitivity [41]. The attention modulates neural activity [42], [43], and a faster time course is allocated for motion processing [44]. However, the result of unpredictable apparent motion did not differ from that for predictable apparent motion. Therefore, prediction and intention as top-down factors have no effect on the results of Experiment 1.

Many researchers have reported that prediction influences the temporal processing of modalities because temporal processing of expected modalities is faster than that of unexpected modalities [45], [46], [47], [48]. However, Spence et al. reported that reaction times for a sensory stimulus followed by another sensory stimulus of the same type were faster than when a cross-modal stimulus was expected [38]. Therefore, Spence et al. suggested that stimulus-driven and expectancy-driven effects must be distinguished in studies of the temporal processing of sensory modalities [38]. The results of Experiments 1 and 3 show no influence on prediction in audiovisual temporal processing during apparent motion perception. Therefore, temporal processing during apparent motion perception may result from stimulus-driven effects rather than expectancy-driven effects. On the other hand, in Experiment 2, although a sensory stimulus was followed by another of the same type of sensory stimulus, audiovisual temporal processing during apparent motion perception differed from that during nonapparent motion perception. This suggests the possibility that despite the stimulus-driven effects with the same type of sensory stimuli, the difference between motion and nonmotion perceptions influenced temporal order perception and the window of temporal integration in audiovisual processing.

Our findings indicate that visual apparent motion affects audiovisual simultaneity perception. For unisensory processing, some researchers have reported that visual motion was perceived more quickly than nonmotion information [49], [50]. However, there remained a need to examine the influence of motion perception on temporal simultaneity perception in multisensory processing. What mechanisms contribute to the finding that apparent motion affects temporal perception? One possibility is the peculiar motion perception mechanisms in humans [51], [52]. Although two discrete stimuli are presented at appropriate spatiotemporal intervals, we can perceive continuous motion. In other words, two discrete stimuli separated by intervals greater than 300 ms are perceived as successive stimuli, whereas if the interval is within a range of 50 to 150 ms, the stimuli are perceived as one moving object [25], [26], [27]. This phenomenon suggests that visual motion perception mechanisms have a binding property that stimuli are perceived as a moving object with spatiotemporal continuity, and a single bounded object is perceived only at certain intervals [20], [53], [54]. Our findings raise the possibility that the binding property in visual motion perception influences audiovisual simultaneity with a brief sound.

Our findings reveal that visual apparent motion affects audiovisual simultaneity by shifting simultaneity toward physical simultaneity, and reduces the temporal window in audiovisual integration. This suggests that visual motion information contributes to accurate perceptions of temporal events in the physical world. In particular, we suggest that the binding property of visual motion perception may affect temporal simultaneity perception in multisensory processing. This binding property will prompt developmental research on motion perception and multisensory integration.

## Supporting Information

Table S1 Results of Experiment 1. A paired t-test is used to compare the results between the apparent motion condition and the normal condition. The table shows the results of paired t-tests of PSSs and JNDs, indicating a significant difference between the TOJ tasks in the apparent motion condition and those in the normal condition.
(DOCX)

Table S2 Results of Experiment 2. A repeated-measures analysis of variance (ANOVA) and multiple comparisons with Holm correction are used to compare the results in the apparent motion condition and those in the successive condition. The table indicates a statistically significant difference between the apparent motion and successive conditions.
(DOCX)

Table S3 Results of Experiment 3. An unpaired t-test is used to compare the results of apparent motion conditions in Experiments 1 and 3. The table shows that unpaired t-tests of PSSs and JNDs revealed no significant difference between the TOJ tasks in the predictable apparent motion condition (Experiment 1) and unpredictable apparent motion condition (Experiment 3).
(DOCX)
