# Supplementary material for: The Effect of Visual Apparent Motion on Audiovisual Simultaneity
Source: PLoS One. 2014 Oct 8;9(10):e110224. doi: 10.1371/journal.pone.0110224 (PMC4190322; doi:10.1371/journal.pone.0110224)
Supplement: Table S1 — Results of Experiment 1. A paired t-test is used to compare the results between the apparent motion condition and the normal condition. The table shows the results of paired t-tests of PSSs and JNDs, indicating a significant difference between the TOJ tasks in the apparent motion condition and those in the normal condition. (DOCX) [file pone.0110224.s001.docx]

| **Table S1.** Results of Experiment 1 | | | |
| --- | --- | --- | --- |
| Participant ID | PSS | | Paired t-test |
|  | apparent motion condition | normal condition |  |
| 1 | -15.3230 | -3.4860 | t(15) = –2.33, *p* = 0.034 |
| 2 | -6.9740 | 7.6236 |  |
| 3 | 19.4694 | 29.9772 |  |
| 4 | 13.0025 | 13.3642 |  |
| 5 | -43.6939 | -10.7020 |  |
| 6 | 15.2174 | 36.3053 |  |
| 7 | -27.0640 | 31.7091 |  |
| 8 | -31.1414 | 17.2117 |  |
| 9 | -5.3711 | -13.2923 |  |
| 10 | -19.2304 | 60.5293 |  |
| 11 | 9.0247 | 37.6906 |  |
| 12 | -8.9263 | -13.2271 |  |
| 13 | 24.1174 | -22.7946 |  |
| 14 | -41.7677 | -37.3585 |  |
| 15 | 27.0163 | 37.0697 |  |
| 16 | 11.0375 | 19.3689 |  |
| Participant ID | JND | | Paired t-test |
|  | apparent motion condition | normal condition |  |
| 1 | 32.6396 | 57.4832 | t(15) = –3.57, *p* = 0.001 |
| 2 | 14.6002 | 47.2173 |  |
| 3 | 33.4595 | 60.9776 |  |
| 4 | 17.3835 | 34.6956 |  |
| 5 | 40.8509 | 94.8784 |  |
| 6 | 30.0467 | 35.6490 |  |
| 7 | 21.3309 | 32.0663 |  |
| 8 | 55.2588 | 55.2861 |  |
| 9 | 44.7801 | 62.8820 |  |
| 10 | 53.0285 | 56.2285 |  |
| 11 | 22.7275 | 30.0972 |  |
| 12 | 12.5701 | 31.2958 |  |
| 13 | 69.2061 | 61.4840 |  |
| 14 | 29.6920 | 26.2714 |  |
| 15 | 15.0903 | 19.8946 |  |
| 16 | 23.4072 | 31.7368 |  |
