# Supplementary material for: The Effect of Visual Apparent Motion on Audiovisual Simultaneity
Source: PLoS One. 2014 Oct 8;9(10):e110224. doi: 10.1371/journal.pone.0110224 (PMC4190322; doi:10.1371/journal.pone.0110224)
Supplement: Table S2 — Results of Experiment 2. A repeated-measures analysis of variance (ANOVA) and multiple comparisons with Holm correction are used to compare the results in the apparent motion condition and those in the successive condition. The table indicates a statistically significant difference between the apparent motion and successive conditions. (DOCX) [file pone.0110224.s002.docx]

| **Table S2.** Results of Experiment 2 | | | | |
| --- | --- | --- | --- | --- |
| Participant ID | PSS | | | Repeated-measures analysis of variance (ANOVA) and Multiple comparisons with Holm correction |
|  | apparent motion condition | successive condition (SOA:300ms) | successive condition (SOA:500ms) |  |
| 1 | -17.0560 | 22.7630 | 32.3063 | F(2, 23) = 15.83, p < 0.001,  apparent motion and an SOA of 300 ms: *p* = 0.042, apparent motion and an SOA of 500 ms: p = 0.012 |
| 2 | -17.3490 | -1.0565 | 3.3593 |  |
| 3 | -3.0524 | -13.2580 | -9.2940 |  |
| 4 | 13.0207 | 47.1110 | 36.7250 |  |
| 5 | -21.9820 | -13.8310 | -14.5791 |  |
| 6 | 27.4401 | 46.5625 | 54.3981 |  |
| 7 | -13.1710 | -4.0706 | 26.8217 |  |
| 8 | -31.9972 | -20.1421 | -18.6447 |  |
| 9 | 21.0251 | 19.0541 | 31.0380 |  |
| 10 | 43.1014 | 62.0973 | 51.6925 |  |
| 11 | -38.9284 | -31.7091 | -29.2002 |  |
| 12 | -25.0991 | -34.5933 | -26.3742 |  |
| Participant ID | JND | | | Repeated-measures analysis of variance (ANOVA) and Multiple comparisons with Holm correction |
|  | apparent motion condition | successive condition (SOA:300ms) | successive condition (SOA:500ms) |  |
| 1 | 23.0291 | 45.8114 | 49.7891 | F(2, 23) = 25.03, p < 0.001,  apparent motion and an SOA of 300 ms: *p* = 0.002, apparent motion and an SOA of 500 ms: p < 0.001 |
| 2 | 32.2172 | 41.5545 | 51.8719 |  |
| 3 | 31.5052 | 32.1539 | 36.0852 |  |
| 4 | 20.9097 | 36.6784 | 38.3342 |  |
| 5 | 38.9102 | 68.3333 | 51.8694 |  |
| 6 | 30.0911 | 43.5283 | 46.8093 |  |
| 7 | 29.5557 | 78.7764 | 69.9001 |  |
| 8 | 34.7315 | 59.8421 | 66.5957 |  |
| 9 | 19.5041 | 22.4218 | 19.1453 |  |
| 10 | 19.9510 | 26.6426 | 26.4635 |  |
| 11 | 12.5759 | 32.0663 | 44.9826 |  |
| 12 | 23.2870 | 38.3181 | 40.3531 |  |
