# Supplementary material for: The Effect of Visual Apparent Motion on Audiovisual Simultaneity
Source: PLoS One. 2014 Oct 8;9(10):e110224. doi: 10.1371/journal.pone.0110224 (PMC4190322; doi:10.1371/journal.pone.0110224)
Supplement: Table S3 — Results of Experiment 3. An unpaired t-test is used to compare the results of apparent motion conditions in Experiments 1 and 3. The table shows that unpaired t-tests of PSSs and JNDs revealed no significant difference between the TOJ tasks in the predictable apparent motion condition (Experiment 1) and unpredictable apparent motion condition (Experiment 3). (DOCX) [file pone.0110224.s003.docx]

| **Table S3.** Results of Experiment 3 | | | |
| --- | --- | --- | --- |
| Participant ID | PSS | | Unpaired t-test |
|  | apparent motion condition  (Experiment 1) | apparent motion condition  (Experiment 3) |  |
| 1 | -15.3230 | 11.6515 | t(26) = –0.11, *p* = 0.92 |
| 2 | -6.9740 | -4.1150 |  |
| 3 | 19.4694 | 11.2804 |  |
| 4 | 13.0025 | -15.8839 |  |
| 5 | -43.6939 | -15.0161 |  |
| 6 | 15.2174 | 0.0000 |  |
| 7 | -27.0640 | 9.6336 |  |
| 8 | -31.1414 | 16.8833 |  |
| 9 | -5.3711 | 39.5334 |  |
| 10 | -19.2304 | -9.4024 |  |
| 11 | 9.0247 | -41.7085 |  |
| 12 | -8.9263 | -51.5592 |  |
| 13 | 24.1174 |  |  |
| 14 | -41.7677 |  |  |
| 15 | 27.0163 |  |  |
| 16 | 11.0375 |  |  |
| Participant ID | JND | | unpaired t-test |
|  | apparent motion condition  (Experiment 1) | apparent motion condition  (Experiment 3) |  |
| 1 | 32.6396 | 36.7931 | t(26) = –0.12, *p* = 0.91 |
| 2 | 14.6002 | 48.5611 |  |
| 3 | 33.4595 | 22.6009 |  |
| 4 | 17.3835 | 41.7005 |  |
| 5 | 40.8509 | 19.6930 |  |
| 6 | 30.0467 | 36.7928 |  |
| 7 | 21.3309 | 34.6852 |  |
| 8 | 55.2588 | 16.7830 |  |
| 9 | 44.7801 | 22.1686 |  |
| 10 | 53.0285 | 22.9245 |  |
| 11 | 22.7275 | 64.2504 |  |
| 12 | 12.5701 | 28.3905 |  |
| 13 | 69.2061 |  |  |
| 14 | 29.6920 |  |  |
| 15 | 15.0903 |  |  |
| 16 | 23.4072 |  |  |
